# Subjective and objective indices in determining stretching effect

**DOI:** 10.1371/journal.pone.0322788

**Published:** 2025-04-29

**Authors:** Shinichi Daikuya, Yumi Okayama

**Affiliations:** Faculty of Health and Medical Sciences, Hokuriku University, Kanazawa, Japan; Fondazione Policlinico Universitario Gemelli IRCCS, ITALY

## Abstract

Stretching is widely used in clinical settings to maintain and enhance physical functions. Despite numerous studies investigating its effects, subjective evaluations by therapists remain common, lacking objective assessment criteria. This study aims to bridge this gap by correlating objective measures with subjective evaluations by therapists and patients. The hypotheses are: (1) significant correlation between objective measures and subjective assessments, (2) consistency in therapist judgments, and (3) identification of objective criteria. This research aims to formalize clinical knowledge and enhance stretching interventions’ applicability. Sixteen physiotherapists participated, with varying clinical experience. Straight leg raising stretching was performed on a simulated patient. Objective measures included joint angle, electromyography, and posterior thigh stiffness. Subjective evaluations were made by therapists and simulated patient using numerical rating scales. Therapists’ ratings correlated positively with straight leg raising angle change and stretching duration. Simulated patient’s ratings correlated positively with an integral electromyographic values of semitendinosus muscle activity but negatively with joint angle change. Regression analysis did not yield significant predictive models. Subjective evaluations may deviate from objective outcomes, highlighting the need for a better understanding of their relationship. Physiotherapists detected small joint angle improvements subjectively, but no objective predictor of this change was found. Patients’ subjective evaluations did not align with objective outcomes. This suggests therapists’ evaluations may be more accurate. Additional investigation into subjective evaluation criteria is warranted. Therapists should be cautious in relying solely on patient feedback, emphasizing the need for objective assessment in clinical practice.

## Introduction

Stretching, a commonly employed technique in various clinical settings, plays a pivotal role in maintaining and enhancing physical and motor functions [1–5]. Numerous studies have investigated the effects of stretching, primarily utilizing indices such as joint range of motion, muscle strength, surface electromyography (EMG), and overall performance [1,6–16].

On the other hand, the effect of stretching in a clinical setting is often judged by subjective indices such as resistance feeling to movement of joints by therapist and/or patient own, palpation of muscles, and physical sensation by the therapist. There have been no studies that aimed to objectively assess the subjective responses of the therapist and compare it with their actual condition in a clinical setting. In addition, there have been no studies that have objectively clarified the criteria for judgment of the therapist.

In view of the above, there are following two major significant gaps in current research related to stretching techniques in clinical settings.

### Objective vs. subjective assessment

Existing studies primarily focus on objective measures like joint range of motion, muscle activity (EMG), and stiffness. However, subjective assessments by therapists, such as resistance felt during joint movement or muscle palpation, are crucial in clinical settings but lack objective validation.

### Lack of integration

There is a notable absence of studies integrating these subjective assessments with objective measures. This gap limits the ability to formalize clinical judgments and criteria used by therapists.

The focus of this study is to bridge these gaps by exploring the relationship between objective indicators (such as joint range of motion, EMG, and stiffness of the target area) measured before and after stretching sessions, and subjective evaluations made by both therapist and simulated patient after stretching sessions. In particular, the purpose of this study is to establish an index that can be readily applied in clinical practice. This study is positioned as a crucial preliminary step toward formalizing the tacit (empirical) knowledge inherent in clinical practice. Thus, the hypotheses for this study are as follows:

1. Objective-Subjective Correlation: We hypothesize that there exists a significant correlation between objective measures (joint range of motion, surface electromyography, and stiffness) and the subjective assessments of therapists and patients regarding the effectiveness of stretching interventions.
2. Consistency in Therapist Judgment: We anticipate that therapists with varying years of experience will exhibit a consistent pattern in their subjective judgments, reflecting a convergence with objective outcomes.
3. Identification of Objective Criteria: It is expected that this study will contribute to identifying and establishing objective criteria for therapist judgments in clinical settings, shedding light on the factors influencing their subjective evaluations.

By addressing these hypotheses, this research aims to provide valuable insights into the interplay between objective and subjective assessments, ultimately enhancing the understanding and applicability of stretching interventions in clinical practice. Therefore, in this study, we examined the relationship among joint range of motion, surface electromyography (EMG), and stiffness of the target area, and subjective judgment of effectiveness by the therapist and the patient before and after stretching, and the duration of SLR stretching.

## Methods

### Subjects

In this study, the results (scores) of each index were tested for differences in five variables before and after stretching: joint range of motion, integral electromyographic values, stiffness of the posterior thigh, and subjective assessments made by both the physiotherapist (subject) and the simulated patient. We estimated the required sample size using SPSS Statistics Ver 28.0.1.0 (IBM), assuming analysis would be conducted via one-way ANOVA. The test power was set at 0.8, the standard deviation at 5, and the mean values of each index between 5 and 10. Based on these parameters, the estimated sample size was determined to be 11.

Subjects were widely recruited by email (1 Aug 2022–31 Oct 2022) for participation among teachers and physiotherapists affiliated with the research institute. Of these, those who gave written consent after receiving a full explanation of the purpose, significance, expected results and risks of the study in writing from the researchers were included in the study.

Based on interviews with applicants for the study, the following were excluded from the study subjects.

1) The person with neurological or orthopedic subjective or objective findings as presenting symptoms
2) The person with a history of neurological disease without any subjective or objective findings as a presenting symptom
3) The person with a history of orthopedic disease without any subjective or objective findings as a presenting symptom

Sixteen physiotherapists without neurological or orthopaedic history or functional abnormalities participated in the study. Their mean age was 29.3 ± 7.0 (22–45) years, and their mean duration of work experience was 77.2 ± 84.3 (5–269) months. In addition, one of the purposes of this study was to examine whether there were differences in the subjective judgments of outcomes by physiotherapists based on their years of clinical experience as physiotherapists. For this reason, the age structure of the physiotherapist group of examinees was as wide as possible, and since a linear study of the relationship must be attempted, a wide distribution of ages was considered.

Prior to the experiment, the purpose, objectives, methods, ethical considerations, and handling of personal information were explained to the subjects orally and in writing, and their consent was confirmed with a signed consent form.

This study was conducted with the approval of the Hokuriku University Research Ethics Review Committee for Human Subjects (Approval No. 2022–5). All study tasks were carried out in accordance with the Declaration of Helsinki. Prior to experiment, according to the decision the Hokuriku University Research Ethics Review Committee for Human Subjects, we explained in advance the outline and invasion of this experiment and the presence/ absence and form of publication, and then conducted subjects who obtained written consent to the purpose of this experiment.

### Implementation

#### Simulated patient.

The simulated patient in this study was one healthy adult male, and all subjects (physiotherapist) were asked to perform stretching on the same simulated patient (one healthy adult male). The reason for this was to avoid differences in the subjective impression (sense of efficacy) of the simulated patients when monitoring their subjective judgment of efficacy when stretching was performed.

#### Stretching.

The therapists performed stretching of hip extensor muscles by straight leg raising (SLR stretching) on supine position according to the method widely practiced in the medical and sports fields. The stretching method, such as intensity and speed of manual manipulation, duration of stretching, physiotherapist’s own posture and so on, were arbitrary methods by individual subject (physiotherapist) in order to make them methods that the individual subject (physiotherapist) themselves was confident could produce results.

**Fig 1:**
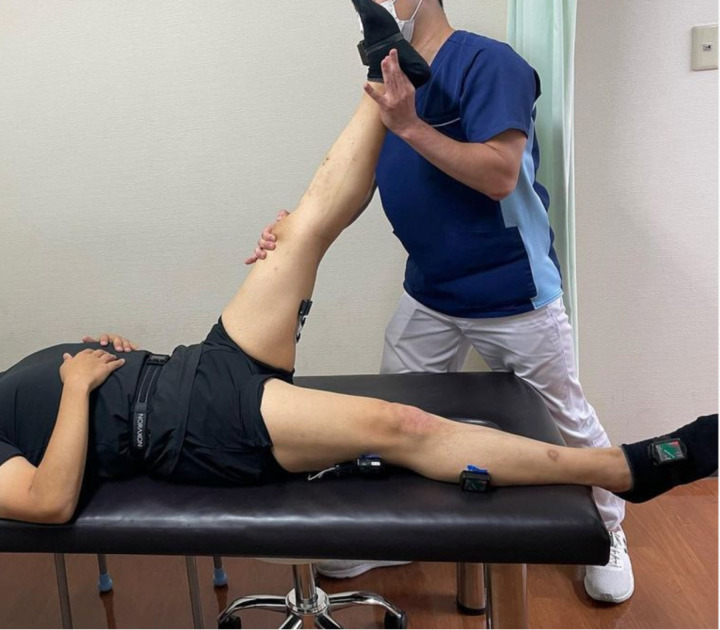
SLR Stretching.

The simulated patient was asked to lie in a supine position on the bed, with the foot to the periphery outside the bed, and in a position as comfortable as possible. No other conditioning was applied to the supine posture of the simulated patient. (Fig 1). The simulated patient was a physiotherapist with more than 30 years of work experience. The same simulated patient was used throughout all experiments. The height of the bed was not adjustable, to ensure the same psychological and physical conditions for the simulated patient during the stretching and when changing posture for objective test and measurement. If the simulated patient was stretched by more than one subject (physiotherapist) on the same day, the interval was at least 20 minutes to eliminate any influence on the range of motion in the previous trials.

#### Measurement of hip flexion angle (SLR angle).

**Fig 2:**
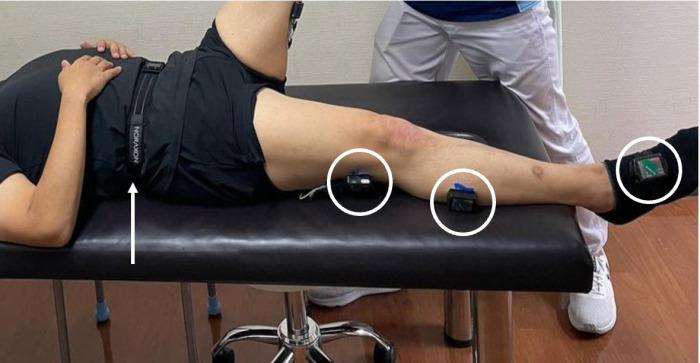
Measurement of Hip Flexion Angle (SLR Angle). The sensors for the lower extremities were placed in the circled areas using double-sided tape. Another sensor for the pelvic girdle was placed in the center of the lumbar back using the belt indicated by the arrow.

In the supine, resting position on the bed, the same examiner held the simulated patient in the knee-extended position in passive maximum hip flexion by the same examiner. Myomotion (NORAXON) was used to measure the hip flexion angle. For measurements by Myomotion, sensors were placed on the pelvic girdle (L5-S1 part) and lower limbs of the simulated patient (Fig 2). In addition, for the sensor of the pelvic girdle (L5-S1 part), it was confirmed that there was no compression in the supine position, and the knee joint position was also monitored simultaneously with the hip flexion angle measurement.

#### Surface electromyography.

Surface electromyography (EMG) data from the biceps femoris and semitendinosus muscles were recorded using Myomotion (Noraxon, USA). The skin was meticulously prepared for analyzing using surface EMG by abrasion with gel and alcohol before electrode placement. Then, pairs of surface electrodes spaced 20 mm apart were placed according to the guidelines for surface EMG for noninvasive assessment of muscles (SENIAM): Biceps femoris; at 50% on the line between the ischial tuberosity and the lateral epicondyle of the tibia, Semitendinosus; at 50% on the line between the ischial tuberosity and the medial epycondyle of the tibia. The electrodes used for our measurements were the disposable type ECG Ambu Blue Sensor M (ref. M-00-S/50, 34mm-40mm). The same examiner performed skin pretreatment and electrode placement, and confirmed that the electrical resistance between electrode and skin was less than 5 kΩ. The recording limb position was the hip in maximum flexion with knee extension before SLR stretching. After SLR stretching, the recording limb position was set the same as before SLR stretching. This position was kept by the same examiner for 10 seconds during which surface electromyography was recorded. The EMG data underwent band-pass filtering (10–500 Hz) before being sampled at 1 kHz. The EMG data were digitized from analog to digital (A/D) using the MyoResearch software (Noraxon, USA) and then imported into a personal computer for further analysis, and Integral electromyographic values (IEMG) during the mid-5 seconds was calculated. IEMG of the biceps femoris and semitendinosus muscles before and after SLR stretching were calculated from the electromyographic waveforms.

#### Posterior thigh stiffness.

**Fig 3:**
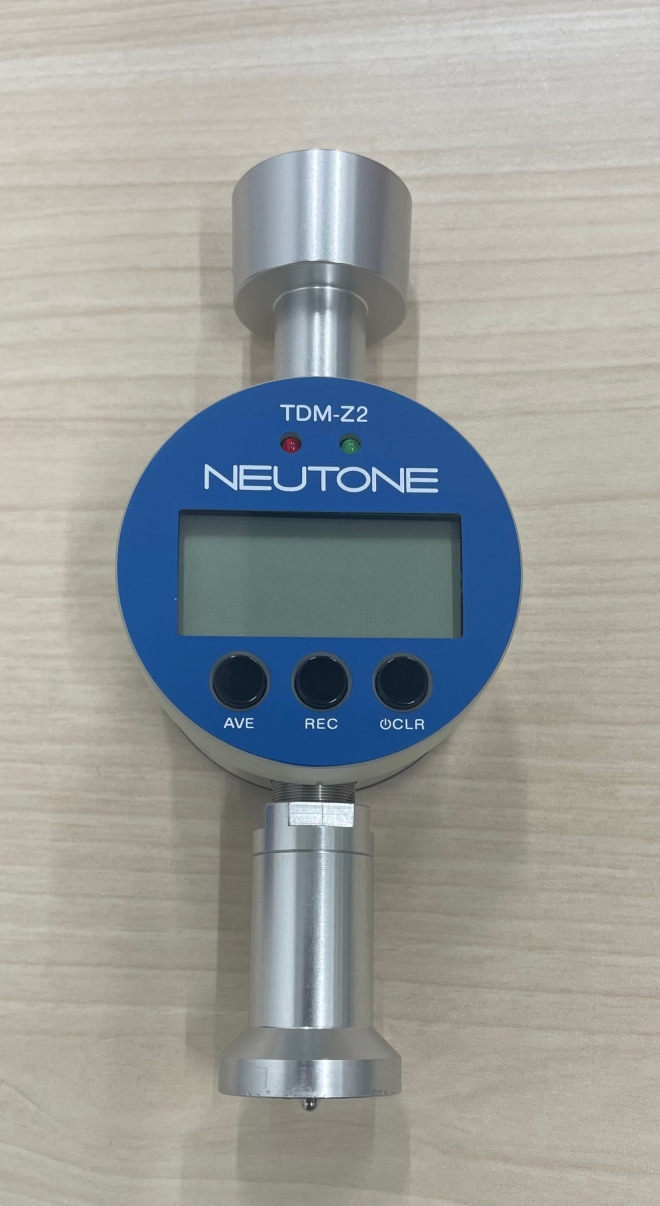
Muscle Hardness Tester.

NEUTONE Muscle Hardness Tester TDM-Z2 (BT) (TRY-ALL) (Fig 3) was used to measure the stiffness of the posterior thigh of the simulated patient. The simulated patient was placed in the resting prone position (foot outside the bed), which had the best reproducibility within the same examiner in the pre-experiment. Throughout all experiments, the same examiner took five measurements, and the average value was calculated. The interval between the five measurements was determined by visually confirming that the skin at the test site had returned to its original state before the next measurement was made. In addition, an algometer is an instrument that applies pressure to the human organism through the skin. The reliability and validity of the algometer have been verified [17,18] examined the validity and reproducibility of the Pressure threshold meter and proved its excellent those quality. The reproducibility and reliability of the Muscle Hardness Tester, which was used in this study, were validated based on the coefficient of variation and intraclass correlation in repeated measurements. It was concluded that the Muscle Hardness Tester is useful for the quantitative evaluation of soft tissue hardness [19].

#### Subjective effect assessed by the subject (physiotherapist) and the simulated patient after SLR stretching.

Self-judgment by the subject (physiotherapist) and the simulated patient was evaluated by applying the NRS [20,21], a method of pain evaluation, as its measure, replacing the pain evaluation with the stretching effect. The 11-point scale was used as the scale. That is, the subjective effect was measured after stretching on an 11-point scale from 0 (no effect at all) to 10 (very effective).

#### Duration of SLR stretching.

The duration of SLR stretching was arbitrary. The same examiner marked on the surface EMG the beginning and end of SLR stretching. The interval between the two points was measured as the duration of SLR stretching.

For SLR angle, IEMG, and Posterior thigh stiffness, the difference between the values before and after SLR stretching was adopted as the result. Pearson’s correlation coefficient, primary regression equation, and coefficient of determination were calculated for the relationship between each of the obtained values using SPSS Statistics Ver 28.0.1.0 (IBM). An analysis of variance and multiple regression analysis were also performed with SLR angle change as the dependent variable and objective measures, excluding the NRS of the subject (physiotherapist) and the simulated patient, as well as the duration of practice experience as forced inputs as independent variables.

## Results

The results for the SLR angle, posterior thigh stiffness, IEMG of the biceps femoris muscle, and IEMG of the semitendinosus muscle, both before and after SLR stretching, as well as the differences between pre- and post-stretching, are presented in Table 1. Additionally, the NRS scores for the physiotherapist (subject) and the simulated patient, as well as the duration of the stretching, are also included in Table 1. SLR angle significantly increased and posterior thigh stiffness significantly decreased before and after stretching. There were no significant changes in other indices before and after stretching. The mean stretching time was 83.6 ± 48.2 (range: 15.0–179.5) seconds.

**Table 1:** Results for each index.

	Before	After	p value	Cohen’s d effect sizes	Differences (After-Before)
SLR angle (degrees)	44.6 ± 2.8 (42.0-52.0)	47.1 ± 2.6 (44.0-55.0)	p < .0001	-1.573	2.6 ± 1.7 (0.0-6.0)
posterior thigh stiffness (points)	37.2 ± 3.8 (33.0-45.0)	36.3 ± 3.2 (33.0-44.0)	p = 0.048	0.537	-0.6 ± 1.9 (-5.0-2.0)
IEMG of biceps femoris (mV • sec)	11.8 ± 2.1 (9.2-16.4)	12.1 ± 2.4 (8.4-16.3)	p = 0.544	-0.155	0.9 ± 1.6 (-3.3-3.7)
IEMG of semitendinosus (mV • sec)	11.4 ± 2.3 (8.1-15.0)	11.7 ± 2.8 (8.3-16.6)	p = 0.660	-0.112	1.1 ± 2.4 (-4.2- 5.5)
NRS of the subject (physiotherapist) (point)	–	4.6 ± 1.6 (2.0-8.0)	p = 0.242	-0.304	–
NRS of the simulated patient (point)	–	5.6 ± 2.8 (0.0-9.0)			–
Duration of SLR stretching (sec)	83.6 ± 48.2 (15.0-179.5)		–	–	–

average ± SD (Min-Max)

The p-value and Cohen’s d effect size were calculated with a paired t-test using SPSS Statistics Ver 28.0.1.0 (IBM).

Table 2 shows the linear regression equations and coefficients of determination for the SLR angle change as the objective variable and the other individual indices as explanatory variables. When the change in SLR angle was used as the objective variable and the other individual indices as explanatory variables, no index was found to be sensitive to the change in SLR angle.

**Table 2:** Linear regression equations and coefficients of determination for SLR change.

		IEMG Change (Biceps)	IEMG Change (Semi-tendinosus)	Posterior thigh stiffness	NRS (Subject)	NRS (Simulated patient)	Duration of SLR stretching
SLR angle Change	Linear regression equation	y = 0.1042x + 2.598	y = -0.0179x + 2.6298	y = -0.2115x + 2.4135	y = 0.4382x + 0.6804	y = -0.479x + 6.7575	y = 0.0241x + 0.505
	Coefficient of determination (R^2^)	0.0106	0.0007	0.0557	0.1745	0.0798	0.4865

The linear regression equations and coefficients of determination for each relationship with SLR angle change as the dependent variable and the other indicators as independent variables. The F value in the analysis of variance was 2.494, with a significance of 0.103. Multiple regression analysis yielded the following equations:


$$\text{Y=0.324}{\text{X}}_{\text{1}}\text{+0.152}{\text{X}}_{\text{2}}\text{+0.028}{\text{X}}_{\text{3}}\text{-0.003}{\text{X}}_{\text{4}}\text{-0.133}{\text{X}}_{\text{5}}\text{+0.498}$$

Y: SLR angle change

X_1_: IEMG change (biceps)

X_2_: Posterior thigh stiffness change

X_3_: Duration of SLR stretching

X_4_: Duration of work experience

X_5_: IEMG change (semitendinosus)

Table 3 shows the correlation coefficients between each indicator with SLR angle change as the dependent variable and the other individual indicators as independent variables. For SLR angle change, a strong positive correlation was found with duration of SLR stretching, and a negative correlation was found with the stiffness change of the posterior thigh. In terms of changes in IEMG, the aspect of the biceps femoris and semitendinosus muscles was linear, and a positive correlation was found between IEMG changes in the semitendinosus muscle and the subject (physiotherapist)‘s NRS; however, no significant relationship with other indices in terms of IEMG was found. The subject (physiotherapist)’s NRS was positively correlated with SLR angle change and duration of SLR stretching. However, the NRS of the simulated patient showed a negative correlation with SLR angle change.

**Table 3:** Correlation coefficients between each indicator.

	IEMG Change (Biceps)	IEMG Change (Semi- tendinosus)	Posterior thigh stiffness	NRS (Subject)	NRS (Simulated patient)	Duration of SLR stretching
SLR angle Change	0.10	−0.03	−0.24	0.42	−0.28	0.70
IEMG Change (Biceps)		0.81	−0.07	0.07	0.08	−0.005
IEMG Change (Semi-tendinosus)			−0.007	0.004	0.306	−0.02
Posterior thigh stiffness				−0.05	−0.08	−0.47
NRS (Subject)					−0.19	0.29
NRS (Simulated patient)						0.006
Duration of SLR stretching						

## Discussion

In subjective evaluation, both patients and physical therapists may overestimate or underestimate the results [22]. Therefore, it is necessary to clarify the relationship between subjective outcome judgments made by physical therapists themselves, outcome judgments made by those who receive physical therapy, and test results as objective outcome judgments in physical therapy situations where there is not always time to make objective 

outcome judgments in clinical practice [22]. In this study, in addition to changes in joint range of motion, surface electromyography, and muscle stiffness as objective evaluation indices, the relationship between these indices and subjective judgments of stretching effect by the subject (physiotherapist) and the simulated patient was examined.

In studies on stretching, range of motion (ROM), and muscle stiffness, a correlation has been reported between an increase in ROM and a decrease in muscle stiffness [23–29]. On the other hand, some reports indicate that while ROM increases with static stretching, no decrease in muscle stiffness was observed [30]. In this study, the results of joint range of motion change before and after SLR stretching indicate that the immediate effect of the SLR stretching performed on the subject (physiotherapist) in this study was small but significant decrease was observed (average: 2.6 degrees). And, the change in joint range of motion after SLR stretching was positively correlated with duration of SLR stretching. Furthermore, the greater the change in joint range of motion, the greater the decrease in stiffness of the posterior thigh, suggesting that SLR stretching was effective. The relationship between the change in SLR angle and the change in stiffness of the posterior thigh suggests that using the stiffness of the center of the posterior thigh as an index to judge the effect of SLR stretching is meaningful when the range of motion of the joint cannot be measured. However, the stiffness of the posterior thigh measured in this study was not a percutaneous measurement of the stiffness of a specific muscle group, and different results may have been obtained if the hardness of the target muscle of SLR stretching had been measured.

In studies on stretching and the IEMG of the target muscle, it has been reported that IEMG decreases during the holding phase of static stretching for 3–5 minutes [27]. Additionally, some studies have reported that inactivation occurs in muscle contractions immediately after prolonged stretching [31], while others have reported that there is no effect on IEMG two hours after static stretching [32]. In this study, although no significant changes were observed in IEMG after stretching, while the IEMG changes of the biceps femoris and semitendinosus muscles were similar. And, only the IEMG change of the semitendinosus muscle was positively correlated with the simulated patient’s NRS. The NRS of the simulated patient was negatively correlated with SLR angle change. These findings suggest that the simulated patient’s subjective sensation may have depended on the sense of extension of the semitendinosus muscle. And since the same simulated patients were used in this study, this impression may be an individual characteristic. It is also possible that the simulated patient may have experienced the effect by another index, such as subjective sensation, which was not addressed in this study. The NRS of the simulated patient did not show any correlation between the SLR angle change and any index other than the IEMG change of the semitendinosus muscle. This indicates that the simulated patient’s sensation was not correlated with the objective data.

Passive resistance during passive joint movement decreases after stretching [24,33]. The subject (physiotherapist)‘s NRS was positively correlated with SLR angle change and SLR stretching duration. The subject (physiotherapist) subjectively perceived an average of 2.6 degrees of angle change. Thus, the subjective interpretation by the subject (physiotherapist) had some credibility even in an environment where it was difficult for the subject (physiotherapist) to use a goniometer, and their perception of the change was less than the measurement granularity (5-degree increments in Japan) in general clinical and educational settings. In this study, while it is considered that the practitioner perceived the decrease in passive resistance to assess the effectiveness of stretching and determine whether to conclude the intervention, the influence of the stretching duration must also be taken into account.

The duration of stretching reported in various studies ranges from short to long, including 30 seconds [29,34,35], 1 minute [23,34–36], 120 seconds [24,34,35], 180 seconds [32], and 5 minutes [23]. There is a report that a longer duration of stretching has a greater effect [28]. In this study, the duration of stretching was set as the time until the subject (physiotherapist) felt that the desired effect had been achieved. As a result, the average duration was 83.6 ± 48.2 seconds, with the shortest being 15 seconds and the longest being 179.5 seconds. While the average duration falls within the range reported by previous studies and is considered effective, a 15-second stretch may be too short to produce actual effects. Also, the SLR angle change was correlated with the duration of SLR stretching in this study. This result supports the findings of Arntz et al., 2023 [28]. However, since the subject (physiotherapist)‘s NRS was correlated with the duration of SLR stretching in this study, subject (physiotherapist)s may have judged the effect of SLR stretching based on the duration of SLR stretching.

In addition, additional tests, such as interviewing subject (physiotherapist) about the basis for their subjective evaluation of NRS, are needed. A follow-up study consisting of interviewing the subject (physiotherapist) about the basis (factors) for their own subjective evaluation criteria is necessary. Thus, our findings suggest that the subject (physiotherapist)’s judgement of the effectiveness of stretching was more accurate than the self-reported effectiveness by the patient.

The results of linear regression equations and coefficients of determination of SLR angle change, direct objective of SLR stretching as the dependent variable, and each of the other measures as independent variables did not yield a significant model for predicting SLR angle change.

Physiotherapists in clinical setting should be aware of the possibility of underestimation or overestimation [22] of their own outcome judgments regarding stretching, while knowing that they are capturing minute changes. In addition, based on the results of this study, judgments regarding the physiotherapy techniques should not rely solely on the patient’s impressions and sensations in clinical practice, since the sensations of the patient being stretched have no relationship to objective findings or the physiotherapist’s judgments.

Finally, the limitations of this study include the following The EMG recordings from the biceps femoris and semitendinosus muscles in this study were not continuously recorded from before to after the stretching, but were recorded before and after the stretching, and were recorded using surface electrodes. Therefore, there is a possibility that the pre-intervention and post-intervention recordings were taken from different sites due to stretching effect of the skin. Furthermore, in this study, the therapists themselves were asked to perform an arbitrary stretching method that they thought would be effective for a time until they judged that it was effective, and the effectiveness of the stretching method was expressed by subjective evaluation. As an ongoing issue derived from the results of this study, it is necessary to verify by what the therapists subjectively judged that the stretching was effective. Specifically, as a continuation of this study, it is necessary to interview the therapists to determine what they judged the subjective effects based on what they felt such as the resistance they felt from their hands (subjected resistance), the weight of the lower limbs during stretching, the speed of the movement to the final range, and the movement and position of the pelvis during the compensations, as factors by which the therapists judged the subjective effects. The interview should be conducted to find out what the subjectively felt effects were based on and how they were judged.

## Conclusion

Physiotherapists subjectively detected joint range of motion improvement (angle difference) of less than 5 degrees. An objective index for predicting the degree of SLR angle change was not found in the present study.

Since the simulated patient judged the effect by an index that was not the change in the index sought by the subject (physiotherapist), our findings suggest that the subject (physiotherapist) should not use the patient’s impressions or sensations as an index of stretching effect.

The results of this study caution against judging the intensity and effectiveness of one’s own treatment techniques based solely on the results of subjective interviews with subjects in the field of physiotherapy, and indicate the need for objective judgment in clinical decision making. The results emphasize the importance of seeking objectivity in physiotherapy intervention and physiotherapy effectiveness without relying on subjective judgments, especially in the education of physiotherapy students and newly graduated physiotherapists.

## Supporting information

S1 File Certification. (PDF)

S2 File PLOSOne Human Subjects Research Checklist. (DOCX)

S1 Fig (TIF)

S2 Fig (TIF)

S3 Fig (TIF)
