# Supplementary material for: Subjective and objective indices in determining stretching effect
Source: PLoS One. 2025 Apr 29;20(4):e0322788. doi: 10.1371/journal.pone.0322788 (PMC12040127; doi:10.1371/journal.pone.0322788)
Supplement: S1 File — (PDF) [file pone.0322788.s001.pdf]

https://www.med-english.com
Translation • Editing • Transcription
Medical English Service
mailto: job@med-english.com

March 1 2023

CERTIFICATE OF PROOFREADING

This is to certify that Medical English Service, a medical editing and translation company established in 1984, edited the following manuscript.

Title: **Subjective and Objective Indices in Determining Stretching Effect**

Authors: **Shinichi Daikuya and Yumi Okayama**

Daniel Mrozek
President
Medical English Service
27 Tachibana-cho Yoshida Sakyo-ku Kyoto, Japan
Tel 81-75-771-4180 Fax 81-75-751-2434

有限会社医学英語総合ビジネス印
