# Supplementary figures and images for: Subjective and objective indices in determining stretching effect
Source: PLoS One. 2025 Apr 29;20(4):e0322788. doi: 10.1371/journal.pone.0322788 (PMC12040127; doi:10.1371/journal.pone.0322788)

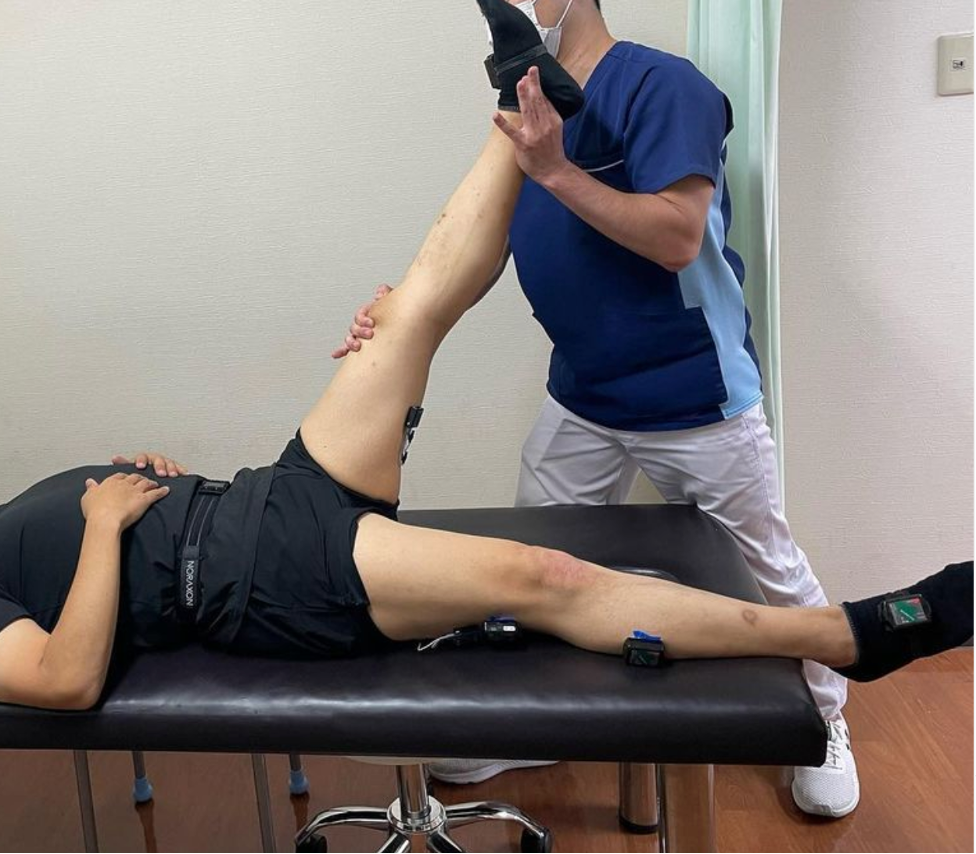

Supplement: S1 Fig — (TIF) [file pone.0322788.s003.tif]

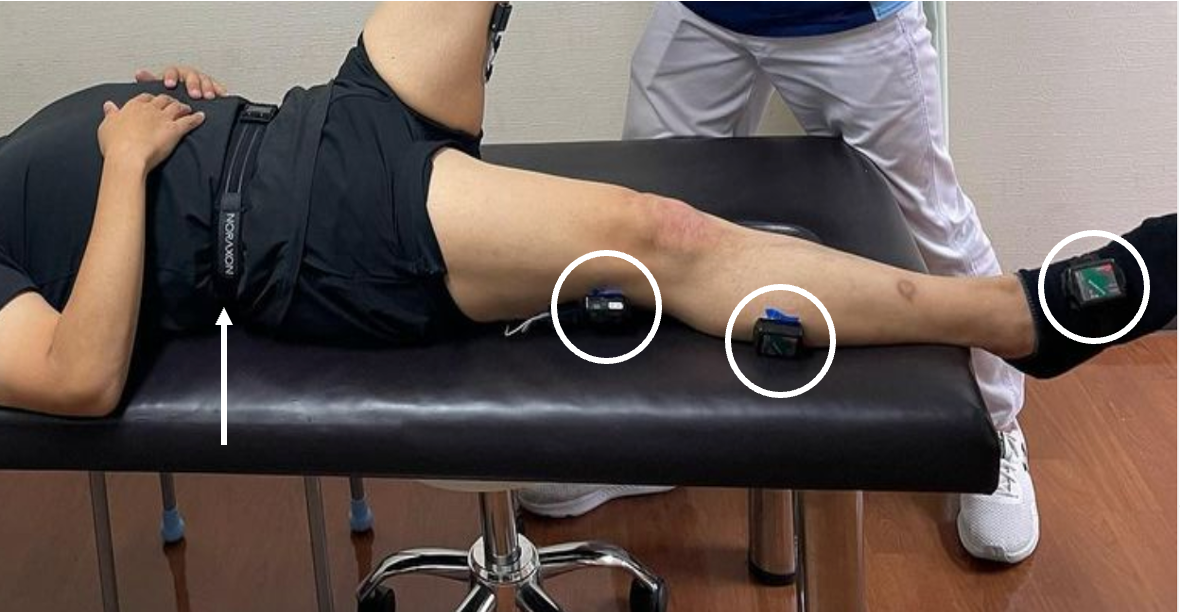

Supplement: S2 Fig — (TIF) [file pone.0322788.s004.tif]

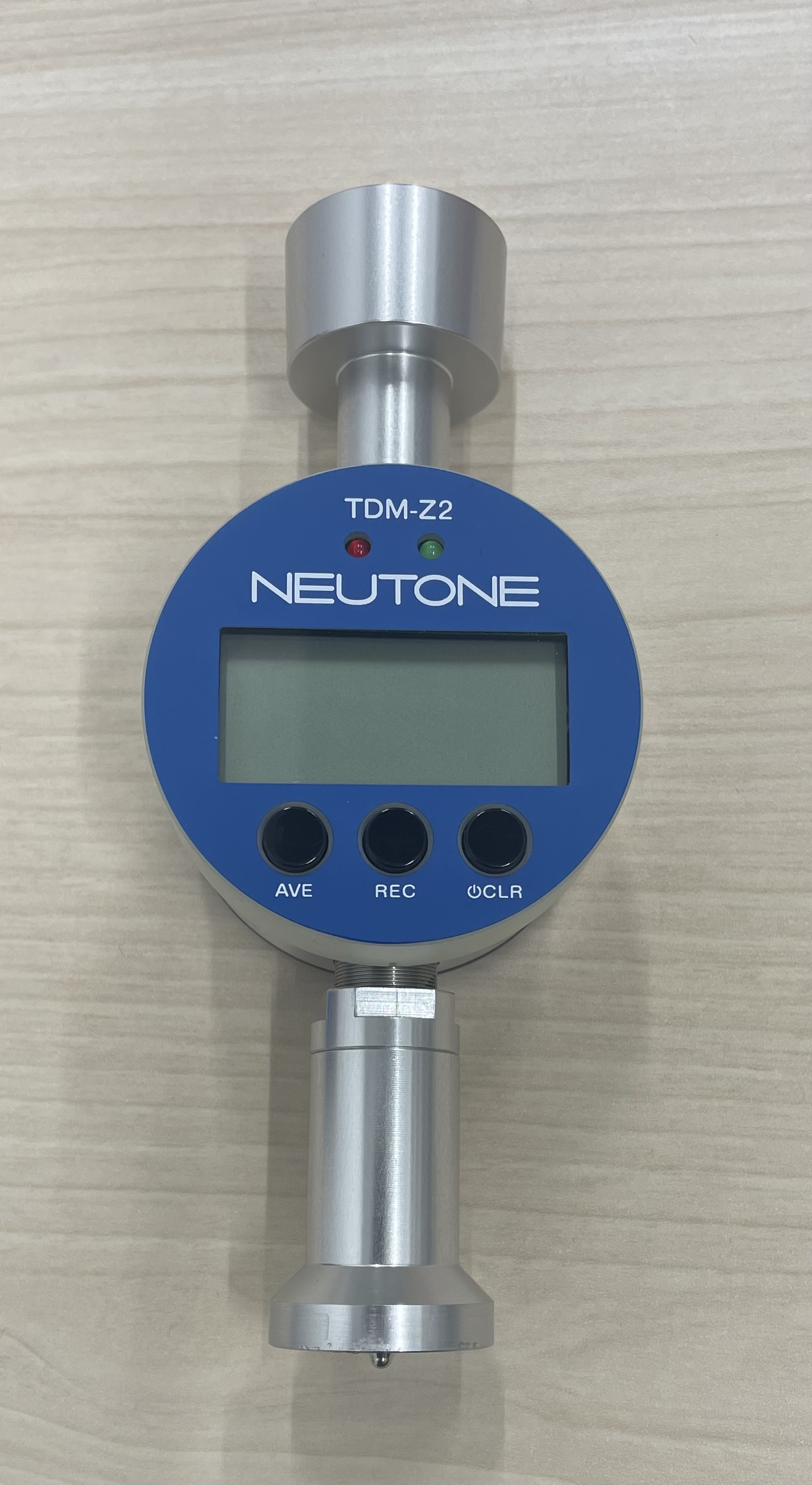

Supplement: S3 Fig — (TIF) [file pone.0322788.s005.tif]
